# Type I insulin-like growth factor receptor over-expression induces proliferation and anti-apoptotic signaling in a three-dimensional culture model of breast epithelial cells

**DOI:** 10.1186/bcr1392

**Published:** 2006-04-03

**Authors:** Gina M Yanochko, Walter Eckhart

**Affiliations:** 1 Molecular and Cell Biology Laboratory, The Salk Institute for Biological Studies, La Jolla, CA 92037, USA

## Abstract

**Introduction:**

Activation of the type I insulin-like growth factor receptor (IGFIR) promotes proliferation and inhibits apoptosis in a variety of cell types. Transgenic mice expressing a constitutively active IGFIR or IGF-I develop mammary tumors and increased levels of IGFIR have been detected in primary breast cancers. However, the contribution of IGFIR activation in promoting breast cancer progression remains unknown. Mammary epithelial cell lines grown in three-dimensional cultures form acinar structures that mimic the round, polarized, hollow and growth-arrested features of mammary alveoli. We used this system to determine how proliferation and survival signaling by IGFIR activation affects breast epithelial cell biology and contributes to breast cancer progression.

**Methods:**

Pooled, stable MCF-10A breast epithelial cells expressing wild-type IGFIR or kinase-dead IGFIR (K1003A) were generated using retroviral-mediated gene transfer. The effects of over-expression of wild-type or kinase-dead IGFIR on breast epithelial cell biology were analyzed by confocal microscopy of three-dimensional cultures. The contribution of signaling pathways downstream of IGFIR activation to proliferation and apoptosis were determined by pharmacological inhibition of phosphatidylinositol 3' kinase (PI3K) with LY294002, MAP kinase kinase (MEK) with UO126 and mammalian target of rapamycin (mTOR) with rapamycin.

**Results:**

We found that MCF-10A cells over-expressing the IGFIR formed large, misshapen acinar structures with filled lumina and disrupted apico-basal polarization. This phenotype was ligand-dependent, occurring with IGF-I or supraphysiological doses of insulin, and did not occur in cells over-expressing the kinase-dead receptor. We observed increased proliferation, decreased apoptosis and increased phosphorylation of Ser^473 ^of Akt and Ser^2448 ^of mTOR throughout IGFIR structures. Inhibition of PI3K with LY294002 or MEK with UO126 prevented the development of acinar structures from IGFIR-expressing but not control cells. The mTOR inhibitor rapamycin failed to prevent IGFIR-induced hyperproliferation and survival signaling.

**Conclusion:**

Increased proliferation and survival signaling as well as loss of apico-basal polarity by IGFIR activation in mammary epithelial cells may promote early lesions of breast cancer. Three-dimensional cultures of MCF-10A cells over-expressing the IGFIR are a useful model with which to study the role of IGFIR signaling in breast cancer progression and for characterizing the effects of chemotherapeutics targeted to IGFIR signaling.

## Introduction

Cycles of proliferation and invasion followed by massive apoptosis are central to the physiology of the mammary gland. The branching ductal architecture develops postnatally in response to hormone stimulation during puberty, but does not reach a fully differentiated state until pregnancy and lactation when hormones stimulate the proliferation and differentiation of terminal ductal lobular units that contain many alveoli. Following lactation, the mammary gland undergoes massive apoptosis and returns to its pre-pregnancy state, a process called involution. Insulin-like growth factor (IGF) signaling is essential for these processes.

Activation of the type I insulin-like growth factor receptor (IGFIR), by binding of IGF-I or supraphysiological doses of insulin, results in receptor autophosphorylation, association and phosphorylation of adapter proteins such as insulin receptor substrate (IRS)-I, Shc, Crk, Grb-2, and Grb-10, and subsequent activation of downstream signaling pathways, including phosphatidylinositol 3' kinase (PI3K)/Akt and Ras/Raf/mitogen-activated protein kinase (MAPK). Activation of the IGFIR leads to proliferation and anti-apoptotic signaling in a variety of cell lines [1,2]. Embryonic mammary buds transplanted from IGFIR knockout mice into syngeneic recipients showed decreased proliferation of cap cells in terminal end buds and decreased branching and extension of ducts into the fat pad [3]. Similarly, IGF-I knock-out mice have fewer terminal end buds and decreased ductal branching [4,5].

Several lines of evidence suggest that the IGFIR is involved in breast cancer progression: breast cancer cell lines express the IGFIR [6]; transgenic mice over-expressing a constitutively active IGFIR develop mammary tumors at an early age [7]; transgenic mice expressing a truncated IGF-I, with decreased affinity for IGF binding proteins, have increased incidence of spontaneous mammary tumors, and tumors appear earlier when combined with p53 mutation [8,9]. IGFIR expression is regulated by transcription factors such as p53 and the estrogen receptor that are mutated in or associated with breast cancer [10-12]. Administration of IGFIR antisense oligonucleotides decreased mammary tumor growth in a mouse model of mammary carcinogenesis [13]. Expression of a dominant negative IGFIR inhibited adhesion and invasiveness of breast cancer cell lines and metastases *in vivo *[14]. Epidemiological studies correlate increased serum IGF-I levels and insulin-like growth factor binding proteins with increased risk of breast cancer [15,16], and increased levels of IGFIR have been found in human breast cancer specimens, although the gene is rarely amplified [17-19]. In addition, the oncogenic activity of IGFIR is well established: NIH3T3 cells expressing IGFIR form colonies in soft agar and induce tumors when transplanted into nude mice [20]. In addition to the receptor, many components of the IGF system have been implicated in breast cancer through epidemiological or cell culture studies [21,22].

Despite this evidence, the contribution of proliferation and survival signaling resulting from IGFIR activation to promote early stages of breast cancer is not well understood. Three-dimensional cultures of mammary epithelial cells form *in vitro *structures with features similar to those of alveolar units of the mammary gland, such as spheroid shape, proliferative arrest, hollow lumen, apico-basal polarity and secretion of basement membrane proteins. Retention of physiological features of the mammary gland while simplifying dissection of the molecular pathways controlling proliferation, apoptosis and extracellular matrix interaction and their alterations by oncogenes make three-dimensional cultures of breast epithelial cells an attractive model to study IGFIR signaling and the processes involved in forming early breast cancer lesions [23-25]. A recent paper by Irie and colleagues [26] demonstrated the isoform specific contribution of Akt to IGFIR acinar structure formation (see Discussion). Here, we demonstrate that IGFIR expression in MCF-10A breast epithelial cells leads to alterations in acinar morphology reminiscent of early stages of breast cancer, namely enlarged, misshapen, filled acinar structures that result from loss of apico-basal polarity and increased proliferation and survival signaling. Inhibition of downstream signaling pathways with UO126 or LY294002 but not rapamycin disrupted IGFIR acinar development.

## Materials and methods

### Cell culture and reagents

MCF-10A cells were obtained from the American Type Tissue Culture Collection and cultured in F12/DMEM (Gibco, Invitrogen Corp., Carlsbad, CA, USA) with 5% horse serum (Gibco), 5 ng/ml epidermal growth factor (Research Diagnostics, Inc., Concord, MA, USA), 10 μg/ml insulin (Research Diagnostics, Inc.), 0.1 μg/ml cholera toxin (Calbiochem, San Diego, CA, USA), 0.5 μg/ml hydrocortisone (Sigma, St. Louis, MO, USA) and penicillin/streptomycin (Gibco). Growth factor reduced Matrigel (BD Biosciences, San Jose, CA, USA) with protein concentrations between 10 and 12 mg/ml was used. IGF-I was from Research Diagnostics, Inc. LY294002 (2-(4-Morpholinyl)-8-phenyl-4H-1-benzopyran-4-one), rapamycin, and UO126 (1,4-Diamino-2,3-dicyano-1,4-*bis*(2-aminophenylthio)butadiene) were from Calbiochem. Antibodies to the carboxyl terminus of the β-subunit of the IGFIR were described previously [27]; extracellular-signal regulated kinase (ERK)2, p70S6 kinase (p70S6K1) and IRS-1 were from Santa Cruz Biotechnology, Inc. (Santa Cruz, CA, USA); phospho-Akt (Ser473), phospho-Akt (Ser473 Immunohistochemistry specific), Akt, phospho-mammalian target of rapamycin (mTOR; Ser2448) and cleaved caspase-3 (Asp175) were from Cell Signaling (Beverly, MA, USA); phospho-ERK1/2 and α-tubulin were from Sigma; laminin V (γ2 chain) was from Chemicon International (Temecula, CA, USA); E-cadherin and Golgi matrix protein of 130 kDa (GM130) were from BD Transduction Laboratories (Franklin Lakes, NJ, USA; Ki-67 was from Zymed Laboratories (San Francisco, CA, USA); and phospho-p70S6K1 (Thr^389^) was from Upstate (Charlottesville, VA, USA). Fluorescently labeled secondary antibodies for immunoblotting were Alexa fluor 680 goat anti-mouse IgG (Molecular Probes, Invitrogen Corp, Carlsbad, CA, USA) and IRDye800 donkey anti-rabbit IgG (Rockland Immunochemicals, Inc., Gilbertsville, PA, USA).

### Retroviral vectors and generation of MCF-10A cell lines

pCLXSN and pCLXSN containing the human IGFIR were described previously [27]. Kinase-dead IGFIR construct (K1003A) was generated by QuickChange (Stratagene, La Jolla, CA, USA) site-directed mutagenesis using the following primers (mutations in bold): sense, 5'-cctgaaaccagagtggccatt**gc**aacagtgaacgag-3'; antisense, 5'-ctcgttcactgtt**gc**aatggccactctggtttcagg-3'. Pooled, stable MCF-10A cell lines expressing the wild-type IGFIR or K1003A were generated using retroviral-mediated gene transfer followed by selection with G418 (400 μg/ml). Transfection of retroviral constructs and vesicular stomatitis virus glycoprotein (VSVG) into human embryonic kidney (HEK)293 cells stably expressing gag and pol produced VSVG pseudotyped retroviruses. Viral supernatant was collected, filtered, supplemented with polybrene (4 μg/ml; Sigma-Aldrich, St. Louis, MO, USA) and added to MCF-10A cells plated at 5 × 10^5 ^cells/10 cm plate the day before infection.

### Immunoblotting (monolayer cultures)

Whole-cell lysates were prepared from monolayer cultures after rinsing cell layers two times with PBS followed by lysis in NP40 buffer (1% NP40, 40 mM Hepes pH 7.5, 120 mM NaCl, 1 mM EDTA, 10 mM pyrophosphate, 10 mM glycerophosphate, 50 mM NaF) supplemented with phenylmethylsulfonyl fluoride, dithiothreitol, and leupeptin just prior to use. Lysates were cleared by centrifugation for 30 minutes at 12,000 rpm at 4°C. Supernatants were collected and Laemmli buffer was added to each sample. Proteins resolved by SDS-PAGE were transferred to Immobilon-FL polyvinylidene fluoride (PVDF; Millipore, Billerica, MA, USA) by semi-dry transfer. Membranes were blocked for 1 hour with 0.1% casein in 0.2× PBS followed by incubation with primary antibodies overnight at 4°C. Secondary antibodies were fluorescently labeled for visualization with an Odyssey infrared imaging system (LI-COR Biosciences, Lincoln, NE, USA).

### Immunoblotting (three-dimensional cultures)

Acinar structures were grown on Matrigel in 35 mm plates. After 16 days of growth, acinar structures were isolated from Matrigel with cold 0.02% EDTA and shaking for 1 hour at 4°C. IGFIR-10A cells isolated from Matrigel were pelleted by centrifugation and lysed as above. Lysates immunoprecipitated with antibodies to p70S6K were separated by SDS-PAGE, transferred to PVDF and immunoblotted with antibodies to p70S6K or phospho-specific antibodies to p70S6K- [T389].

### Morphogenesis assay

Three-dimensional cultures of MCF-10A cells were grown in 8-well chamber slides (Falcon) as described [28] using growth factor reduced Matrigel (BD Biosciences) with protein concentration between 10 and 12 mg/ml. Except where indicated, assay medium (F12/DMEM, supplemented with 2% horse serum, 0.5 μg/ml hydrocortisone, 0.1 μg/ml cholera toxin, 2% Matrigel, 5 ng/ml epidermal growth factor, 10 μg/ml insulin or 10, 50 or 100 ng/ml IGF-I, pen/strep) was changed every 3 to 4 days. Inhibitors were added after allowing acinar development to progress for three days. Growth medium containing inhibitors was replaced every 2 to 3 days.

### Immunofluorescence analysis and image acquisition

MCF-10A acinar structures were immunolabeled as described [28] with the following modifications: permeabilization with 0.5% Triton X-100 was performed for 30 to 40 minutes at room temperature to avoid Matrigel solubilization at 4°C. Nuclei were counterstained with TO-PRO-3 iodide (TO-PRO-3; Molecular Probes) prior to mounting with Gel/Mount (biømeda, Foster City, CA, USA) or Pro-Long Gold Antifade (Molecular Probes). Images were obtained with a Zeiss LSM 5 scanning confocal microscope or a Leica SP2 AOBS confocal microscope, converted to JPEG (Zeiss LSM 5 images) and arranged using Adobe Photoshop 7.0.

## Results

**Figure 1:**
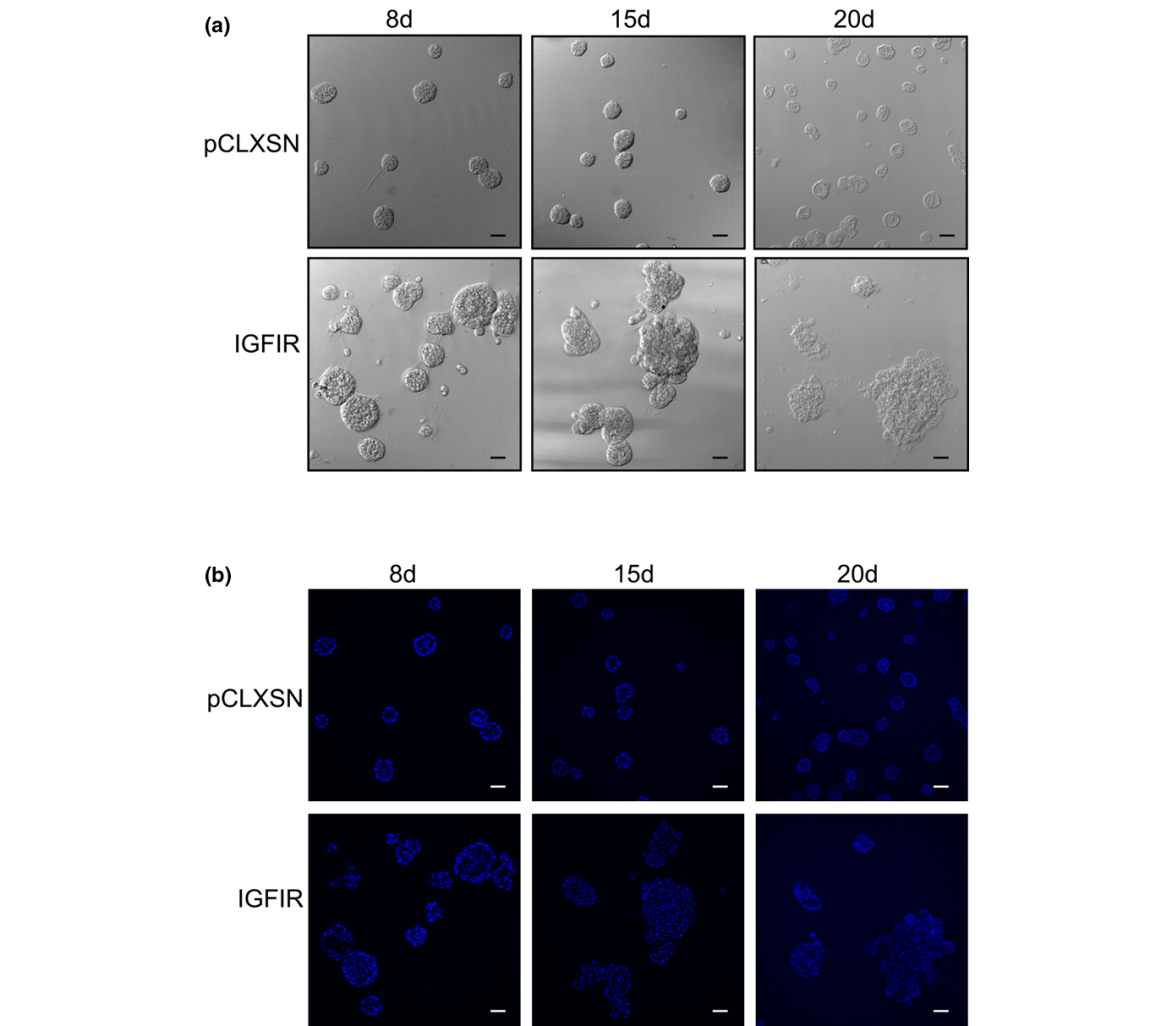
MCF-10A cells over-expressing the type I insulin-like growth factor receptor (IGFIR) form large, misshapen, filled acinar structures. **(a) **Differential interference contrast images of acinar structures formed from stable pools of MCF-10A cells infected with virus expressing empty vector (pCLXSN; upper panels) or wild-type IGFIR (lower panels) after 8, 15 and 20 days of growth. Scale bars = 50 μm. **(b) **Equatorial confocal sections of corresponding images in (a). Control (pCLXSN; upper panels) and IGFIR structures (lower panels) were labeled with the nuclear counterstain TO-PRO-3 after 8, 15 and 20 days of growth in three-dimensional culture. Scale bars = 50 μm.

During the initial phases of MCF-10A acinar development (days 0 to 4) a single cell plated on basement membrane (Matrigel) progresses to a cluster of cells at which time polarization and survival signaling in cells in contact with basement membrane drive the formation of a polarized outer layer of cells. Apoptosis and autophagy clear the inner cells, those that lack contact with the basement membrane, to generate a hollow, polar, growth arrested structure by approximately day 10 [29-31]. To study the effects of IGFIR activation on acinar morphogenesis, stable pools of MCF-10A cells expressing the wild-type human IGFIR or empty vector, pCLXSN (hereafter called control), were generated by retroviral infection. Figure 1 shows differential interference contrast (DIC) and confocal sections of control and IGFIR structures after 8, 15 and 20 days. Nuclei were labeled with the nuclear stain TO-PRO-3. Consistent with results reported previously [29,30], control cells form round acinar structures that have reached their maximal size by day 8. Luminal cells are observed in control structures at day 8, but by day 15, control structures had hollow lumina, a feature that was retained with successive days in culture (Figure 1b). In contrast, although IGFIR structures appear round after 8 days of acinar development, they are larger than the control structures. By day 15, IGFIR structures are noticeably misshapen (Figure 1a) and, unlike control structures, there is no noticeable lumen formation (Figure 1b). The IGFIR structures remain large, misshapen and filled even when cultured up to 40 days (data not shown). These results were confirmed with three independently derived stable pools of cells and three lots of Matrigel.

**Figure 2:**
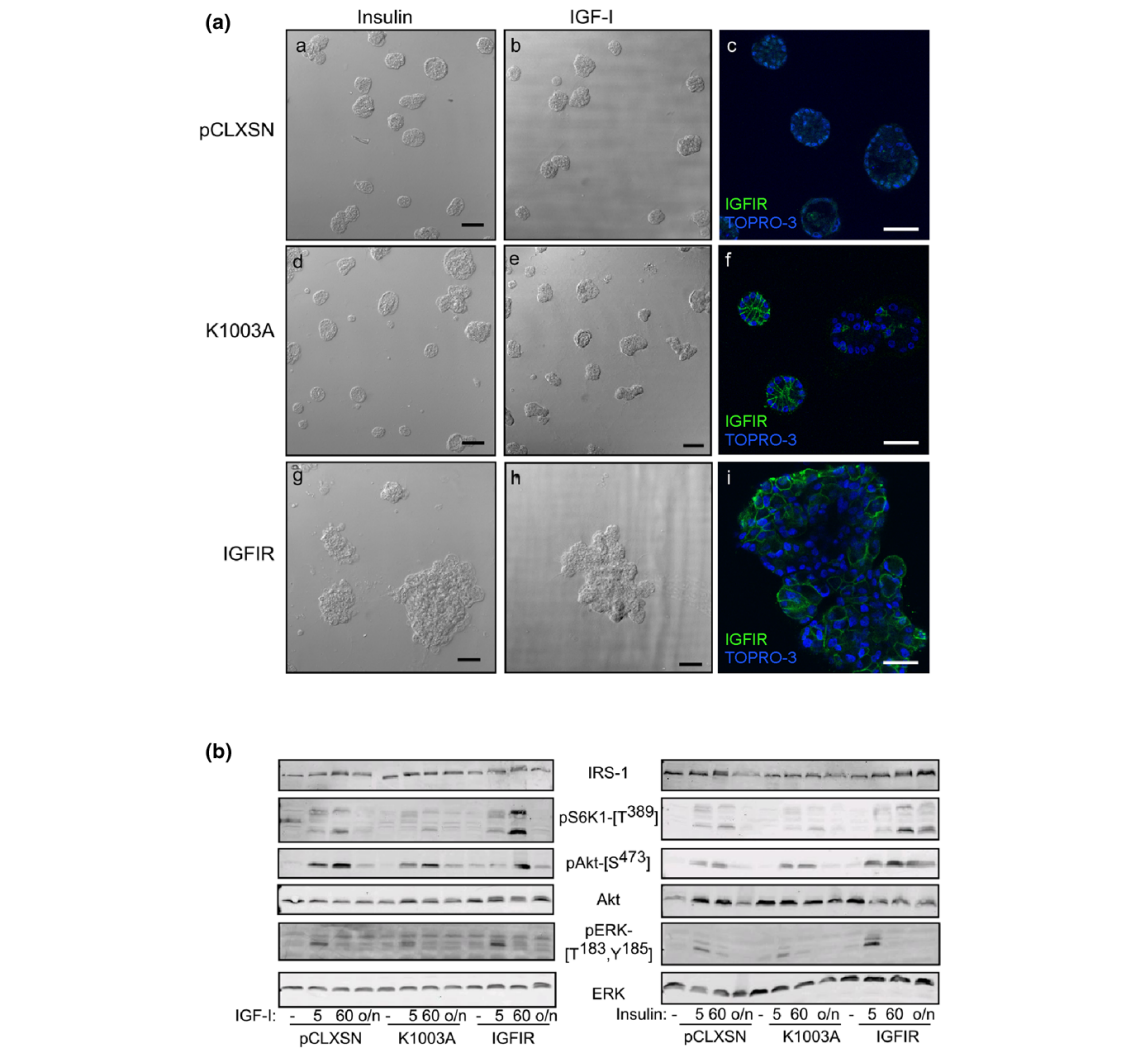
Lack of acinar overgrowth in MCF-10A cells expressing kinase-dead IGFIR. **(a) **Differential interference contrast images of acinar structures formed from stable pools of MCF-10A cells infected with virus expressing empty vector (pCLXSN; panels a, b), kinase-dead (K1003A; panels d, e) and wild-type IGFIR (panels g, h); scale bar = 80 μm. Equatorial confocal sections of pCLXSN (panel c), K1003A (panel f) and wild-type IGFIR (panel i) acinar structures labeled with antibodies to the IGFIR (green) and the nuclear counterstain TO-PRO-3 (blue); scale bar = 40 μm. Cells were grown for 20 days in media supplemented with 10 μg/ml insulin (panels a, c, d, f, g, i) or 100 ng/ml IGF-I (panels b, e, h). **(b) **Western blots of whole-cell lysates from monolayer cultures of MCF-10A cells expressing empty vector (pCLXSN), kinase-dead (K1003A) or wild-type IGFIR (IGFIR) untreated or treated with 50 ng/ml IGF-I (left panel) or 10 μg/ml insulin (right panel) for 5 minutes, 60 minutes or overnight (o/n) after overnight incubation in serum-free media. Proteins were separated by 12.5% SDS-PAGE, transferred to PVDF and incubated overnight with the indicated antibodies to signaling proteins downstream of IGFIR activation.

To test whether these phenotypes were dependent on the protein kinase activity of IGFIR, we generated stable pools of MCF-10A cells expressing a kinase-dead IGFIR construct, with lysine 1003 in the ATP-binding site mutated to alanine (K1003A) [32]. Figure 2a shows DIC images and confocal sections through control, K1003A and IGFIR structures after 20 days in three-dimensional culture grown in the standard three-dimensional growth medium (see Materials and methods), which contains 10 μg/ml insulin (Figure 2a, panels a, c, d, f, g, i) or with 100 ng/ml IGF-I substituted for insulin (Figure 2a, panels b, e, h). At 10 μg/ml (about 2 μM), insulin is acting through the IGFIR. While IGF-I, insulin or IGF-II can activate the IGFIR, the affinity of IGF-I for the IGFIR is three-fold greater than that of insulin or IGF-II [33,34]. Whether cultured in the presence of insulin or IGF-I, control structures were small and round (Figure 2a, panels a, b). Regardless of activation by insulin (Figure 2a, panel g) or IGF-I (Figure 2a, panel h), wild-type IGFIR structures were large and misshapen while K1003A structures had the small, round appearance of control structures. Confocal cross-sections of acinar structures labeled with antibodies to the IGFIR and the nuclear counterstain TO-PRO-3 (Figure 2a, panels c, f, i) showed that, unlike control, IGFIR structures had filled lumina (Figure 2a, panel i), similar to structures formed from MCF-10A cells over-expressing activated Akt1, ErbB2, colony-stimulating factor receptor (CSF1R) or human growth hormone [29,30,35,36]. K1003A structures formed lumina and only the smallest structures had detectable IGFIR expression (Figure 2a, panel f). As expected, cells within IGFIR structures had IGFIR expression at the plasma membrane (Figure 2a, panel i), whereas IGFIR expression was below detection in control structures (Figure 2a, panel c).

We performed western blots on whole-cell lysates from monolayer cultures of control, K1003A and IGFIR-10A cells treated with insulin (10 μg/ml) or IGF-I (50 ng/ml). Figure 2b shows that IGFIR-10A cells have increased response to insulin and IGF-I compared to control cells as assessed by phosphorylation of p70S6K at Thr389, phosphorylation of Akt at Ser^473 ^and phosphorylation of ERK (Thr183 and Tyr185). Treatment of K1003A-10A cells with insulin or IGF-I did not augment phosphorylation of downstream signaling pathways; however, there was still some detectable phosphorylation of Akt and ERK after insulin and IGF-I stimulation, most likely resulting from the mixed population of infected cells.

**Figure 3:**
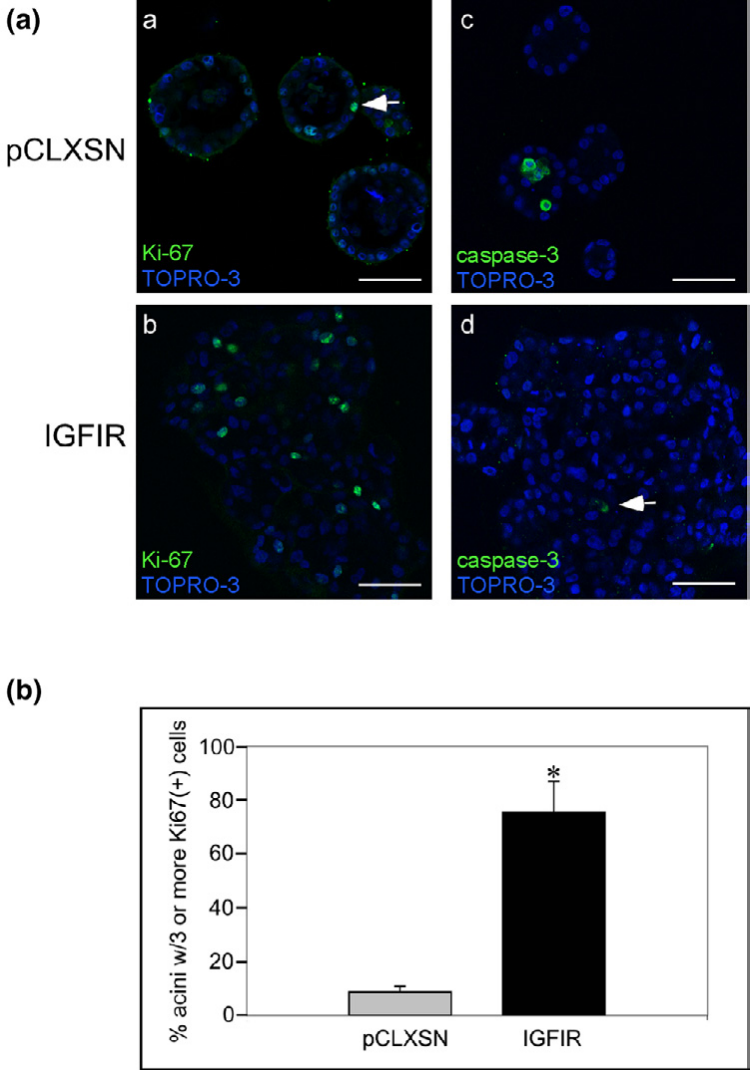
Increased proliferation and decreased luminal apoptosis in IGFIR structures. **(a) **Confocal sections of 34-day acinar structures from control (pCLXSN; panels a, c), and IGFIR-expressing (panels b, d) MCF-10A cells labeled with antibodies to the proliferative marker Ki-67 (green; panels a, b) or cleaved caspase-3 (green; panels c, d) and the nuclear counterstain TO-PRO-3 (blue). Scale bars = 50 μm. Arrows indicate a Ki-67-labeled cell (panel a) and a cleaved caspase-3 labeled cell (panel d). **(b) **Bar graph showing the percent of acini with three or more Ki-67-positive cells in control and IGFIR-expressing structures. Data are mean ± standard deviation from three different experiments grown for 20 days from two independently derived cell lines. At least 100 acini were counted in each experiment; the asterisk indicates significance, *p* < 0.01, Student's *t *test.

Figure 3a shows representative, equatorial confocal sections through acinar structures formed from control and IGFIR-10A cells grown for 34 days and labeled with antibodies to Ki-67, a marker of proliferation (Figure 3a, panels a, b) and cleaved caspase-3, a marker of apoptosis (Figure 3a, panels c, d). As expected after 34 days in culture, control cells had undergone proliferative arrest and the few Ki-67-labeled cells were restricted to the outer cell layer in contact with the basement membrane (Figure 3a, panel a, arrow). In contrast, IGFIR structures had many more Ki-67 positive cells and these were found throughout the acinar structures (Figure 3a, panel b). We confirmed this observation by counting structures with at least three Ki-67-labeled cells in control and IGFIR structures from three independent experiments. After 20 days in culture, significantly more IGFIR structures had three or more proliferating cells (75 ± 12%) compared to control structures (9 ± 2%, *p* < 0.01, Student's *t *test; Figure 3b).

In addition to increased cell proliferation, IGFIR structures displayed decreased cell death. Panels c, d of Figure 3a show representative cross sections of control and IGFIR structures labeled with antibodies to cleaved-caspase-3 after 34 days in culture. In control structures, apoptosis and autophagy clear the lumen of acinar structures by approximately day 10 of acinar development [28]. The lumina of control structures were hollow and any remaining cells in the lumen were undergoing apoptosis after 34 days in culture (Figure 3a, panel c). However, the lumina of IGFIR structures remained filled with cells at this time point, few of which had detectable cleaved caspase-3 (Figure 3a, panel d).

**Figure 4:**
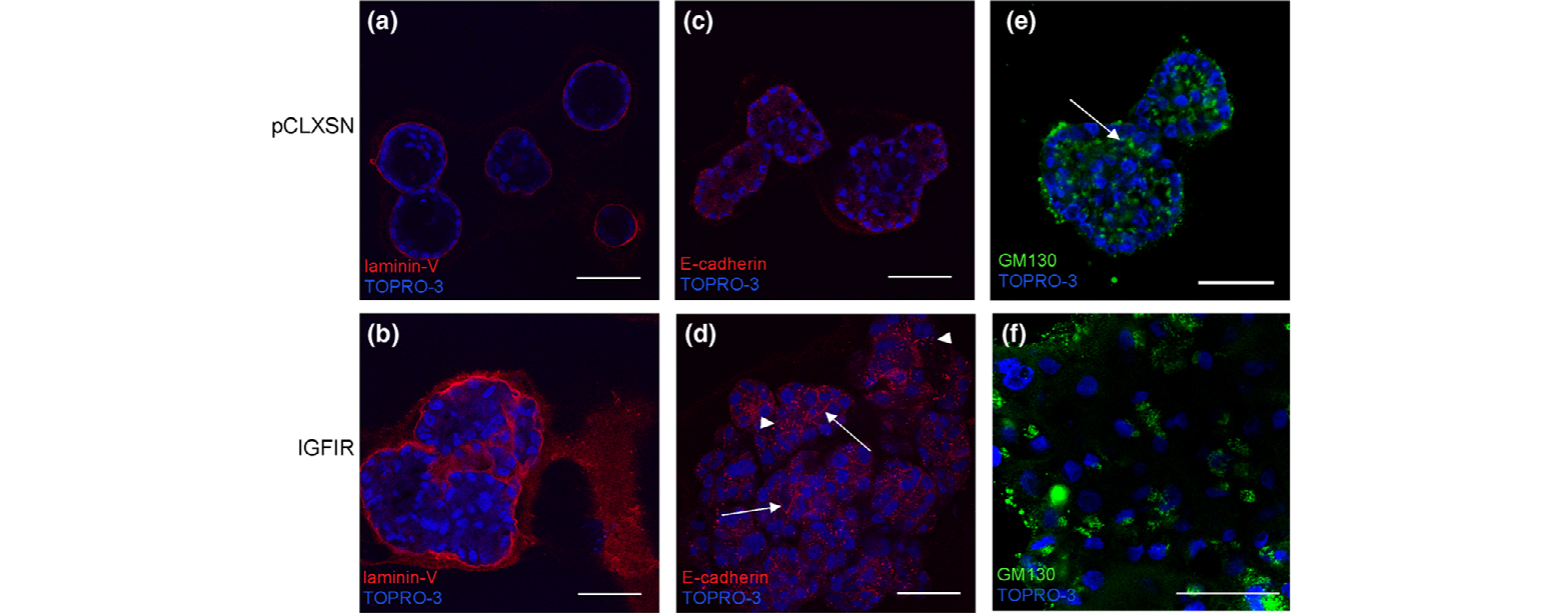
Disrupted apico-basal polarity of IGFIR structures. Equatorial confocal sections of 20-day control (pCLXSN, panels a, c, e) and IGFIR structures (panels b, d, f) labeled with antibodies to laminin V (red; panels a, b), E-cadherin (red; panels c, d) and Golgi matrix protein of 130 kDa (GM130; green; panels e, f) and the nuclear counterstain TO-PRO-3 (blue). Arrows indicate localization of E-cadherin at cell-cell junctions (panel d) and apical localization of GM130 in control structures (panel e). Arrowheads show sites of punctate E-cadherin labeling (panel d). Scale bars = 50 μm.

MCF-10A acinar structures exhibit polarized organization with the apical surface located toward the lumen and basal surfaces in contact with basement membrane [28-30]. We observed polar organization of control structures with basal deposition of laminin V (Figure 4, panel a), E-cadherin oriented basally along lateral membranes (Figure 4, panel c) and apical orientation of GM130, a golgi protein, in outer cells (Figure 4, panel e, arrow). In contrast, cells within IGFIR structures retained features of epithelial cells but their organization was disrupted. Laminin V was generally secreted basally but there were areas of laminin V within IGFIR structures (Figure 4, panel b). E-cadherin was localized at sites of cell junctions (Figure 4, panel d, arrows) but we also observed areas of punctate E-cadherin labeling (Figure 4, panel d, arrowheads), suggesting internalization of E-cadherin and possible changes in cell adhesion within IGFIR structures. However, we did not observe changes in total E-cadherin levels (data not shown). Disruption of E-cadherin localization, with areas of both punctate and labeling at cell junctions, was observed in acinar structures formed by MCF-10A cells over-expressing both CSF1R and CSF-1 [32]. GM130, a golgi protein apically located in outer cells of control structures, lacked distinctive localization and was found throughout IGFIR structures (Figure 4, panel f).

**Figure 5:**
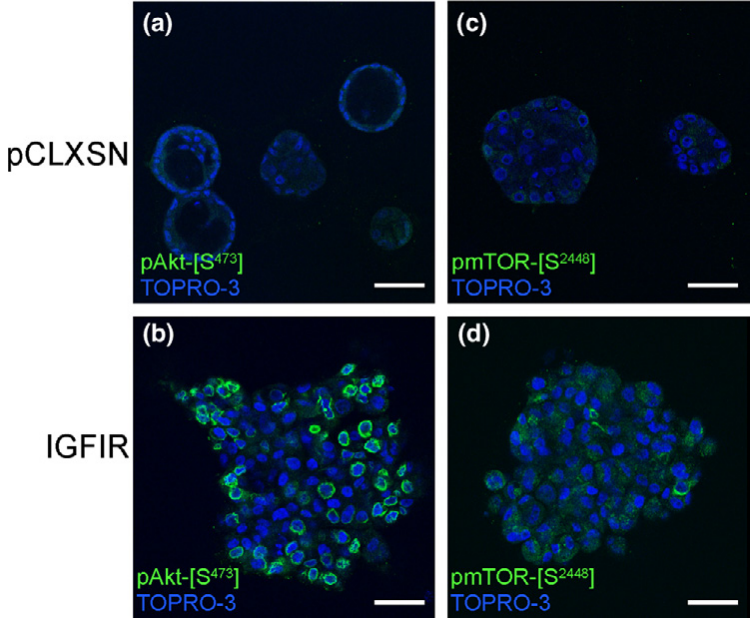
Induction of Akt signaling in IGFIR structures. Equatorial confocal sections of 34-day control (pCLXSN, panels a, c) and IGFIR acinar structures (panels c, d) labeled with phosphospecific antibodies to Akt- [S473] (green; panels a, b) and mammalian target of rapamycin (mTOR)- [S2448] (green; panels c, d) and the nuclear counterstain TO-PRO-3 (blue). Scale bars = 50 μm.

Activation of the IGFIR results in phosphorylation of the beta subunit, subsequent phosphorylation and recruitment of adapter proteins such as IRS-I and Shc, and activation of two main signal transduction pathways: PI3K/Akt and Ras/Raf/MAPK. Figure 5 demonstrates activation of signaling molecules downstream of IGFIR. ERK phosphorylation in IGFIR-10A cells is transient in response to insulin or IGF-I stimulation in monolayer culture, with maximal activation after five minutes (Figure 2b). We were consistently unable to detect phosphorylated ERK in IGFIR structures by immunofluorescence (data not shown) despite being able to detect it in MCF-10A structures over-expressing Raf (Dr Gray Pearson, personal communication). However, IGFIR activation of MAPK is likely to be weak compared to over-expressed Raf.

In response to growth factors, Akt, downstream of PI3K, is phosphorylated at two sites, Thr308 and Ser473, and contributes to growth and survival signaling by phosphorylating downstream targets such as mTOR, BAD (Bcl-x_L_/Bcl-2-associated death promoter homolog) and the forkhead transcription factors (reviewed in [37,38]). We analyzed the role of Akt activation in survival signaling in IGFIR structures by labeling with antibodies specific for Akt phosphorylated on Ser473, indicative of Akt activation [39]. In control structures, we observed phosphorylated Ser473 of Akt only in cells in contact with the basement membrane and only in early stages of acinar development (Figure 5, panel a). However, phosphorylated Akt was observed throughout IGFIR structures even at late time points (Figure 5, panel b), suggesting a critical role for Akt signaling in the growth and survival signaling of IGFIR acinar structures. We used antibodies specific for mTOR phosphorylated on Ser2448 to monitor mTOR activation in IGFIR structures. Although the identity of the kinase responsible for phosphorylation of Ser2448 following growth factor stimulation is unclear, Ser2448 phosphorylation is generally accepted to be indicative of mTOR activation [40-43]. We observed phosphorylation of Ser2448 of mTOR in cells throughout IGFIR structures (Figure 5, panel d) but not in those of control structures (Figure 5, panel c).

**Figure 6:**
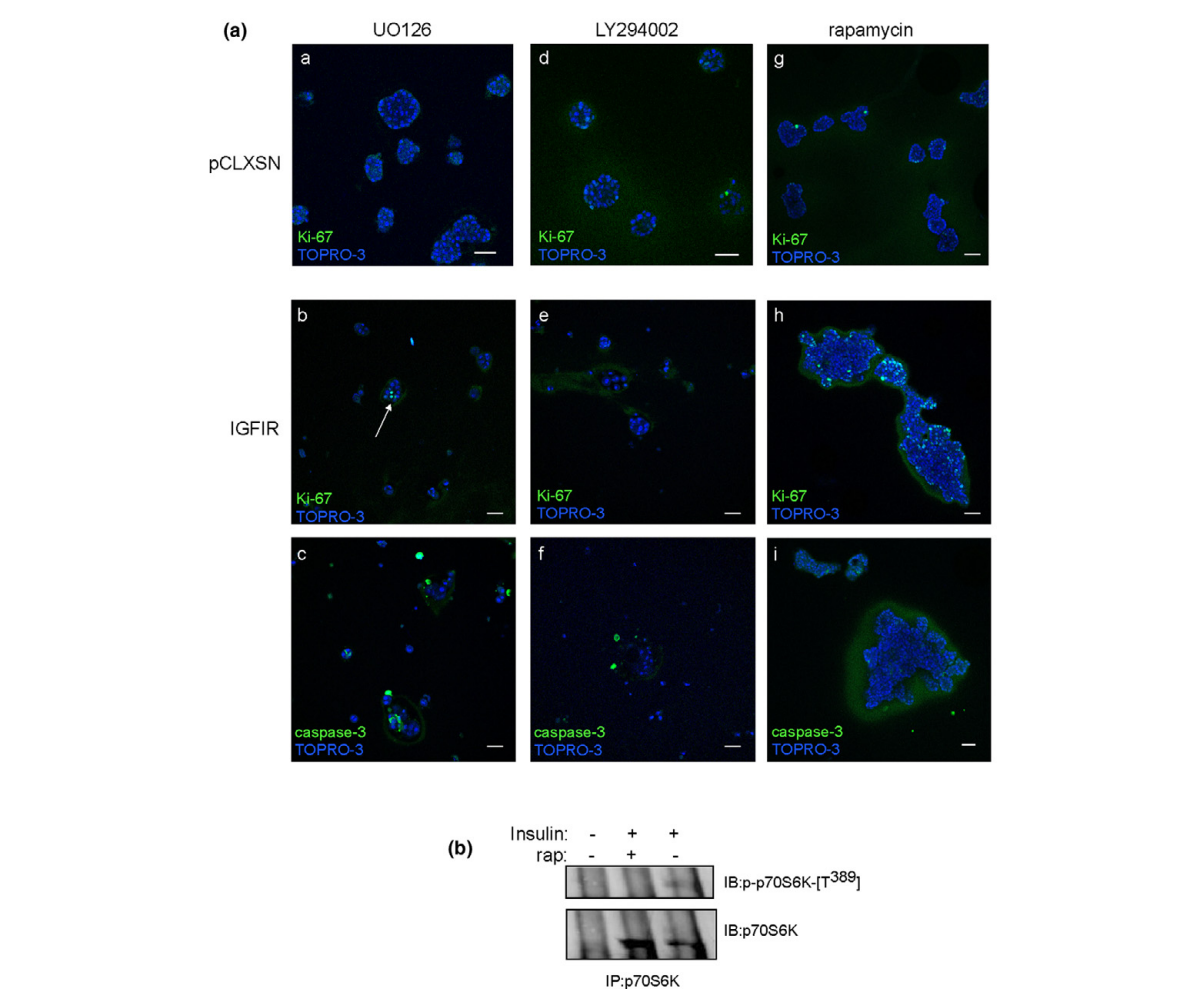
Inhibition of PI3K or MEK but not mTOR disrupts IGFIR acinar development. **(a) **Confocal sections of 20-day control (pCLXSN, panels a, d, g) and IGFIR (panels b, c, e, f, h, i) acinar structures grown in the presence of the MEK inhibitor, UO126 (10 μm; panels a-c), the PI3K inhibitor LY294002 (50 μm; panels d-f) and the mTOR inhibitor rapamycin (40 nM; panels g-i). Fixed structures were labeled with the nuclear counterstain TO-PRO-3 (blue) and with antibodies to Ki-67 (green; panels a, b, d, e, g, h) or cleaved caspase-3 (green; panels c, f, i). Scale bars = 50 μm. The arrow in panel b indicates Ki-67-labeled cells. **(b) **Whole-cell lysates of 16d IGFIR acinar structures were isolated after overnight serum starvation followed by 30 minute pretreatment with DMSO (vehicle) or 40 nM rapamycin and subsequent 30-minute incubation with 10 μg/ml insulin. Lysates were immunoprecipitated with antibodies to p70S6K, separated by 12.5% SDS-PAGE, transferred to PVDF and immunoblotted with antibodies to p70S6K or phosphospecific antibodies to Thr389 of p70S6K as indicated.

We tested the involvement of the PI3K/Akt and the Ras/Raf/MAPK pathways in promoting proliferation and survival signaling in IGFIR structures by pharmacological inhibition of PI3K with 50 μM LY294002 [44,45], MAP kinase kinase (MEK) with 10 μM UO126 [46], and mTOR with 20 to 100 nM rapamycin [47,48]. IGFIR-10A and control cells were grown in three-dimensional cultures with standard growth medium for three days to allow initiation of acinar development, and then inhibitors were maintained in the growth medium during all subsequent media changes for an additional 12 to 15 days (see Materials and methods). Figure 6a shows confocal sections of IGFIR (panels b, c, e, f, h, i) and control structures (panels a, d, g) grown in the presence of UO126 (panels a-c), LY294002 (panels d-f), and rapamycin (panels g-i). Cultures were labeled with the nuclear counterstain TO-PRO-3 and antibodies to Ki-67 (Figure 6a, panels a, b, d, e, g, h) and cleaved caspase-3 (panels c, f, i).

Inhibition of MEK with 10 μM UO126 prevented any significant development of IGFIR structures. Any structures present in these cultures appeared as clusters of cells (Figure 6a, panels b, c). There was no apparent lumen formation, but we were able to detect both Ki-67 labeled cells (Figure 6a, panel b, arrow) and cleaved caspase-3 labeled cells (Figure 6a, panel c) in IGFIR structures with UO126 treatment; 10 μM UO126 is not generally cytotoxic as no effect was observed on the development of control structures (Figure 6a, panel a). The lack of appreciable structure formation suggests an important role for MAPK activation in early stages of IGFIR structure development.

Inhibition of PI3K with 50 μM LY294002 had a similar effect on IGFIR structure formation: any structures present appeared as clusters of cells (Figure 6a, panels e, f). There was no apparent lumen formation but, unlike with UO126 treatment, we did not detect any Ki-67-labeled cells (Figure 6a, panel e). Although rare, we could detect cells undergoing apoptosis in cultures treated with 50 μM LY294002 as assessed by cleaved-caspase-3 labeling (Figure 6a, panel f); 50 μM LY294002 was not generally cytotoxic, as no effect on control structure formation was observed (Figure 6a, panel d). These results highlight the importance of PI3K and MEK activation in early stages of IGFIR structure formation and suggest that IGFIR-10A cells have become physiologically dependent on IGFIR signaling.

Three-dimensional cultures of MCF-10A cells expressing myristoylated Akt formed large, partially filled structures with increased proliferation at early stages of growth [47]. Rapamycin (5 and 20 nM) prevented overgrowth induced by Akt activation, suggesting a critical role for mTOR in mediating the growth promoting effects of Akt [47]. To determine the contribution of raptor/mTOR signaling following Akt activation in IGFIR structures, we treated developing acinar structures with rapamycin [48]. Unexpectedly, rapamycin had no apparent effect on the growth of IGFIR structures; IGFIR structures remained large and misshapen in the presence of 40 nM rapamycin (Figure 6a, panels h, i) and in concentrations up to 100 nM (data not shown). In addition, we consistently observed Ki-67 positive cells throughout the structures, including cells lacking contact with the basement membrane (Figure 6a, panel h). Similar to untreated or DMSO-treated (data not shown) IGFIR structures, few apoptotic cells were observed (Figure 6a, panel i). Rapamycin did not affect the formation of control structures (Figure 6a, panel g) but was effective in inhibiting insulin-induced phosphorylation of Thr389 of p70S6 kinase after insulin-induced stimulation of day 16 IGFIR acinar structures (Figure 6b). These results suggest that while Akt signaling is upregulated in IGFIR structures, the hyperproliferation phenotype and anti-apoptotic signaling were not mediated solely through Akt activation of raptor/mTOR signaling.

## Discussion

Breast cancer is a progressive disease: pre-invasive lesions, such as atypical hyperplasias and carcinomas *in situ*, have increased cell numbers and filled and enlarged mammary alveoli that remain contained by basement membrane proteins; cancerous cells overcome the constraints of the basement membrane and invade the surrounding connective tissue in invasive stages; advanced, metastatic stages are characterized by cancer cells leaving the breast, usually via the lymphatic system, and invading other organs [49]. Three basic processes are abnormally regulated in this progression: cellular proliferation, survival signaling and invasion. Dysregulation of these processes are features of all cancers and can be recapitulated with *in vitro *models [50]. Recapitulation of normal mammary tissue with model systems, such as three-dimensional cultures of breast epithelial cells, provides an attractive system with which to determine the effect of oncogenic disruption of proliferation, survival signaling and invasion on breast epithelial cell biology without additional complications such as inflammatory and hormone signaling [25].

Our data support and extend those reported in a recent paper by Irie and colleagues [26] demonstrating isoform specific effects of knocking down Akt1 or Akt2 in IGFIR acinar structures. The general morphological features of IGFIR-10A cells grown on matrigel (increased proliferation, decreased apoptosis, misshapen and filled acinar structures) are similar to those observed by Irie and colleagues with cells grown on a 50:50 mixture of Matrigel/collagen. We showed that IGFIR structures had disrupted organization while retaining epithelial characteristics: laminin V secretion basally as well as within IGFIR structures; GM130 localization no longer apically oriented; and disruption of E-cadherin localization but no changes in total amounts of E-cadherin. These results support the conclusion by Irie and colleagues that IGFIR cells have not undergone a complete epithelial-mesenchymal transition. In addition, although not discussed in their paper, they show disorganized labeling of α 6-integrin that corresponds with what we observed with laminin V labeling of IGFIR structures. Knockdown of both Akt1 and Akt2 resulted in acini that were severely disrupted, supporting our data showing that inhibition of PI3K with LY294002 prevents significant acinar formation of IGFIR but not control cells and confirms the importance of both isoforms for the IGFIR acinar phenotype. In addition, Irie and colleagues showed that knock-down of Akt2 prevented overgrowth of IGFIR structures and resulted in slightly larger but otherwise normal acinar structures. Knock-down of Akt1 in IGFIR cells resulted in a dramatic phenotypic change: invasive structures, epithelial-mesenchymal transition and increased migration in a trans-well assay. These changes resulted from loss of inhibition of ERK by Akt1.

Acinar structures formed from MCF-10A cells expressing a variety of oncogenes have several characteristic features: increased proliferation, decreased apoptosis, and filled lumina. Activation of ErbB2 in three-dimensional cultures of MCF-10A cells resulted in highly proliferative, multi-acinar structures with filled lumina [29]. These structures retained certain features of epithelial cells, such as secretion of basement membrane proteins, but lacked the apical-basal polar organization typical of MCF-10A cells. Constitutive activation of the CSF-1 receptors through an activated receptor construct or via autocrine production of CSF-1 by MCF-10A cells also resulted in highly proliferative acinar structures, yet they lacked the multi-acinar organization of ErbB2 structures and instead exhibited Src-dependent disruption of cell-cell junctions such that individual cells were capable of dissociating from an acinus [35]. Over-expression of activated Akt1 in MCF-10A cells resulted in enlarged acini that were only moderately filled; apoptosis was not completely inhibited as both viable and apoptotic cells could be found within the lumina and proliferation was enhanced only in early days of acinar development [47]. Rapamycin treatment of Akt1 structures prevented the increased cell size, overgrown, and filled structures, indicating that Raptor/mTOR signaling was mediating the effects of Akt1 in these cells [47]. Finally, expression of human growth hormone in MCF-10A cells also resulted in enlarged, disorganized and filled acinar structures with increased proliferation and decreased apoptosis [36]. Growth hormone acting through IGF-I is essential for proper mammary gland development [4,51-53].

Acinar structures formed from IGFIR-10A cells display notable similarities and differences with each of these phenotypes. First, IGFIR structures displayed increased proliferation as assessed by Ki-67 expression, similar to ErbB2, CSF1R and growth hormone structures. However, the increase in proliferation in IGFIR structures occurs during both early (data not shown) and late stages of acinar development, as opposed to structures formed from over-expression of Akt1 in which proliferation is seen only in early stages. Second, IGFIR structures had decreased apoptosis, assessed by cleaved caspase-3 expression, as would be expected from their filled lumina, a feature that is similar to ErbB2, CSF1R, Akt1 and growth hormone structures. The anti-apoptotic effects could occur through direct actions on apoptotic signaling molecules, such as through suppression of Bim [45] or represent cross-talk between the Ras/Raf/MAPK and PI3K/Akt signaling pathways in these cells. Third, IGFIR structures retain some features of epithelial cells, such as secretion of certain basement membrane proteins, but, similar to ErbB2 and growth hormone-expressing MCF-10A cells, they appear to have disrupted apico-basal polar organization. It is this last feature that may distinguish the phenotype of IGFIR, ErbB2 and growth hormone-expressing structures from that seen with simultaneous expression of proliferative and anti-apoptotic proteins: expression of both cyclin D1 and Bcl-xL, or human papilloma virus 16 E7 and Bcl2, results in only slightly enlarged and modestly filled acini that retain apico-basal polarity [30].

Over-expression of IGFIR in a breast cancer cell line, MCF-7, and growth on Matrigel resulted in enlarged structures as a result of aggregation of cells due to increased cell-cell adhesion via E-cadherin [54]. Addition of 20 ng/ml IGF-I stimulated aggregation of MCF-7 cells even in the absence of over-expressed IGFIR. The ability of MCF-7/IGFIR cells to grow for prolonged periods in this system (20 days) was enhanced with increasing levels of receptor expression. These results highlight several important differences between MCF-7 and MCF-10A cells used to determine the effect of IGFIR over-expression. First, MCF-10A cells are a spontaneously immortalized cell line and though they have genotypic changes (such as myc amplification and loss of p16) that may make them more susceptible to phenotypic changes resulting from introduction of an oncogene, they do not form colonies in soft agar or tumors in nude mice, unlike MCF-7 cells. Second, unlike MCF-10A cells, MCF-7 cells express the estrogen receptor. Given the cross-talk between estrogen receptor and IGFIR signaling, this may represent an important difference in the ability of IGFIR over-expression to promote cell aggregation in one system and stimulate proliferation and survival signaling in another, although it is not clear to what extent proliferative and survival signaling were stimulated in structures formed from MCF-7 cells over-expressing the IGFIR.

Dependence on the continued signaling of a mutated or over-expressed oncogene may be exploited for targeted cancer therapies [55]. Antisense oligonucleotides to Ki-ras decreased growth of pancreatic cell lines expressing mutated but not wild-type Ki-ras [56]. Inhibition of MEK selectively affected cells and tumors with mutated BRAF compared to wild-type or RAS mutant cells due to unique ERK-dependent cyclinD1 expression in BRAF mutant cells [57]. Rather than induce normal acinar structures, inhibition of PI3K or MEK with LY294002 and UO126, respectively, completely disrupted acinar development of IGFIR structures. No detectable effect of PI3K or MEK inhibition was observed on development of control acini, suggesting that MCF-10A cells over-expressing the IGFIR have become physiologically dependent on IGFIR signaling and that chemotherapeutics targeted to the IGFIR may selectively affect cancerous cells with elevated IGFIR signaling.

Rapamycin prevented acinar morphology associated with over-expression of activated Akt1 and mutated PI3K [47,58]. Although Akt is activated in IGFIR structures, rapamycin was not effective in preventing the growth of IGFIR structures, despite its ability to prevent insulin-induced phosphorylation at Thr389 of p70S6K (Figure 6b). Several possible reasons exist for this apparent discrepancy. First, signaling via raptor/mTOR may not be a primary pathway driving proliferation and survival signaling in IGFIR structures. Activation of signaling pathways in addition to Akt/mTOR may be sufficient to override the contribution of raptor/mTOR to proliferation and survival signaling. Second, rapamycin treatment may inhibit the negative feedback loop through mTOR/p70S6K to IRS-1 (reviewed in [59,60]). Our unpublished data suggests that this is at least partly responsible. Third, the recent paper by Irie and colleagues [26] showed that knock-down of Akt2 but not Akt1 in IGFIR-10A cells resulted in slightly larger, but otherwise normal acinar structures. Since rapamycin inhibited the abnormal growth of Akt1-10A cells but does not inhibit IGFIR-10A cells, this may represent additional isoform specific signaling differences, with Akt1 potentially selectively activating raptor/mTOR. The lack of effectiveness of rapamycin in preventing IGFIR acinar phenotype may have important implications on the usefulness of mTOR as a target for breast cancer therapeutics and suggests that rapamycin analogues may be best used in combination with other therapeutics [61,62].

Epithelial cell proliferation, survival signaling and invasion of the surrounding stromal tissue are critical processes for normal mammary gland development, suggesting a strong link between normal mammary development and pathogenesis of breast cancer. The increased proliferative and survival signaling we observed in IGFIR structures are compatible with the established role of IGF-I signaling in mammary gland development. IGFIR knockout mice die shortly after birth due to respiratory failure, but transplanted embryonic mammary buds from IGFIR knockout mice in syngeneic recipients showed decreased proliferation of cap cells in the terminal end buds and decreased branching and extension of ducts into the fat pad [3]. Similarly, IGF-I knock-out mice had fewer terminal end buds and decreased ductal branching [4,5]. Mice expressing a truncated human IGF-I that has reduced affinity for insulin-like growth factor binding proteins had persistent, progressive ductal hypertrophy after several rounds of lactation, decreased natural involution and three-fold increase in the incidence of spontaneous mammary tumors [8,9]. Other growth factors involved in mammary gland development have also been implicated in the pathogenesis of breast cancer. For example, the tyrosine kinase receptor ErbB2, amplified in 30% of breast cancers, is important for early steps in terminal end bud formation and ductal extension into the mammary fat pad of mice, and MCF-10A cells expressing activated ErbB2 form multi-lobed, filled acinar structures [29,63,64].

## Conclusion

Several factors promoting or affecting breast cancer pathogenesis, such as Her2/neu, estrogen and progesterone receptors, E-cadherin, vascular endothelial growth factor, and BRCA1 have been identified and are useful as prognostic indicators or therapeutic targets [65-67]. In this work we demonstrate that increased IGFIR signaling promotes morphological and growth phenotypes reminiscent of early pathological changes in breast cancer. Our results extend the well-known importance of IGFIR activation for proliferative and survival signaling. This model system will be useful for identifying factors that augment IGFIR signaling to enhance breast cancer progression as well as for characterizing novel chemotherapeutics working through the IGFIR signaling pathway.

## Abbreviations

CSF = colony-stimulating factor; CSF1R = colony-stimulating factor receptor; DIC = differential interference contrast; DMEM = Dulbecco's modified Eagle's medium; DMSO = dimethyl sulfoxide; ERK = extracellular-signal regulated kinase; GM130 = Golgi matrix protein of 130 kDa; IGF-I = type I insulin-like growth factor; IGFIR = type I insulin-like growth factor receptor; IRS-I = insulin receptor substrate I; MAPK = mitogen-activated protein kinase; MEK = MAP kinase kinase; mTOR = mammalian target of rapamycin; p70S6K = p70S6 kinase; PBS = phosphate-buffered saline; PI3K = phosphatidylinositol 3' kinase; TO-PRO-3 = TO-PRO-3 iodide.

## Competing interests

The authors declare that they have no competing interests.

## Authors' contributions

GMY participated in the experimental design and interpretation of results, carried out the experimental procedures and drafted the manuscript. WE participated in the experimental design, interpretation of results and revision of the manuscript.
